# Dengue Epidemiology in Mexico: Temperature as a Contributing Factor to National Dengue Trends

**DOI:** 10.3390/diseases14040133

**Published:** 2026-04-07

**Authors:** Juan Manuel Bello-López, Dulce Milagros Razo Blanco-Hernández, Andres Emmanuel Nolasco-Rojas, Emilio Mariano Durán-Manuel, Víctor Hugo Gutiérrez-Muñoz, Carol Vivian Moncayo-Coello, Jesus Alberto Meléndez-Ordoñez, José Alberto Díaz-Quiñonez, Magnolia del Carmen Ramírez-Hernández, Adolfo López-Ornelas, María Concepción Tamayo-Ordóñez, Yahaira de Jesús Tamayo-Ordóñez, Francisco Alberto Tamayo-Ordóñez, Benito Hernández-Castellanos, Luis Gustavo Zárate-Sánchez, Oscar Sosa-Hernández, Julio César Castañeda-Ortega, Claudia Camelia Calzada-Mendoza, Alejandro Cárdenas-Cantero, Clemente Cruz-Cruz, Miguel Ángel Loyola-Cruz

**Affiliations:** 1 Hospital Juárez de México, Mexico City 07760, Mexico; 2 Sección de Estudios de Posgrado e Investigación, Escuela Superior de Medicina, Instituto Politécnico Nacional, Mexico City 11340, Mexico; 3 Escuela de Medicina y Ciencias de la Salud, Tecnológico de Monterrey, Ciudad de México 14380, Mexico; 4 Hospital Nacional Homeopático, Hospitales Federales de Referencia, Mexico City 06800, Mexico; 5 Laboratorio de Ingeniería Genética, Departamento de Biotecnología, Facultad de Ciencias Químicas, Universidad Autónoma de Coahuila, Saltillo 25280, Mexico; 6 Centro de Biotecnología Genómica, Instituto Politécnico Nacional, Reynosa 88710, Mexico; 7 Facultad de Química, Universidad Autónoma del Carmen, Ciudad del Carmen 24180, Mexico; 8 Facultad de Biología, Universidad Veracruzana, Circuito Gonzalo Aguirre Beltrán s/n, Zona Universitaria, Xalapa 91090, Mexico; 9 Dirección General de Epidemiologia, Mexico City 01480, Mexico; 10 Centro Universitario de Ciencias de la Salud, Universidad de Guadalajara, Guadalajara 44340, Mexico

**Keywords:** dengue, climatic change, vector borne disease

## Abstract

The increasing burden of dengue represents a growing global public health concern. Among the factors associated with rising dengue incidence, climate change, particularly increasing temperatures, has been frequently highlighted, alongside other environmental, biological, and social determinants. The emergence of dengue in previously non-endemic areas and its sustained increase in incidence have become increasingly common in recent decades. Objective: The aim of this study was to describe national dengue case trends in Mexico from 1990 to 2023 and to assess their association with temperature over the same period using a descriptive, retrospective analysis of epidemiological surveillance and temperature data. Methods: Epidemiological data on confirmed dengue cases and incidence were obtained from the Morbidity Yearbook of the General Directorate of Epidemiology (DGE) of the Mexican Ministry of Health. These data were used to construct epidemic curves and to analyze the geographic distribution of incidence using quartiles. Temperature data were derived from the national annual mean calculated from monthly reports issued by the National Water Commission (CONAGUA). Associations between temperature and dengue cases and incidence were explored over the study period. Results: Temporal analysis revealed a significant increase in both dengue cases and incidence in Mexico, with a positive association with temperature during the same period. Quartile-based geographic analysis showed that state-level classifications remained relatively stable across periods, with several states clustering within or tending toward the group considered endemic. Conclusions: The results of this study show an increase in cases and incidence of dengue over time, as well as a positive association between cases/incidence of dengue in Mexico and the increase in the national average temperature during the study period; however, due to its descriptive and retrospective design, causal inference is not possible. Dengue transmission is inherently multifactorial, and the observed trends likely reflect the combined influence of climatic conditions, historical expansion of transmission cycles, vector establishment, and unmeasured socio-epidemiological factors. The absence of entomological indicators, additional climatic variables, and spatially or seasonally disaggregated analyses limits the ability to capture localized dynamics. Overall, temperature should be interpreted as a contributing factor within a complex system rather than as the sole driver of dengue trends, underscoring the need for integrated surveillance and control strategies in both endemic and non-endemic regions.

## 1. Introduction

Climate change is recognized as an urgent threat to global public health by the World Health Organization (WHO) [1]. In 2023, a series of climatic and political events propelled the global agenda on climate and health [2,3]. Climate change is a problem that directly affects human pathogenic diseases; it has been reported that 58% of infectious diseases affecting humanity worldwide have been exacerbated by climate change [4]. One of the most evident consequences of climate change is the sustained increase in global temperature [5], which has also influenced the frequency of intense rainfall, agricultural droughts, and ecological imbalances [6]. Changes in environmental conditions directly affect the transmission and incidence of diseases caused by infectious microorganisms or transmitted by vectors [7]. Increasing climate variability generates a cascade of risks that may increase the number of cases of infectious diseases such as dengue or allow their emergence in regions where they were previously absent [5,6,7,8]. For this reason, it is essential that health systems be prepared to respond adequately to these emerging threats.

Dengue is a viral disease caused by a single-stranded RNA flavivirus transmitted primarily by mosquitoes of the *Aedes* genus, particularly *Aedes. aegypti* and *Aedes. albopictus* [9]. The dengue virus exists as four immunologically distinct serotypes (DENV-1, DENV-2, DENV-3, and DENV-4). Infection with one serotype generally confers lifelong immunity against that specific serotype but only temporary and partial protection against the others; consequently, subsequent infections with different serotypes may increase the risk of more severe clinical manifestations. These immunological dynamics contribute to the complex epidemiological patterns of dengue and may influence the occurrence and magnitude of epidemics independently of environmental factors [10,11,12,13]. Dengue is currently considered one of the most important vector-borne threats to global public health. Globally, the incidence has increased substantially, and it is estimated that approximately 50% of the world’s population lives in areas at risk. The WHO reported a sustained increase in reported cases between 2000 and 2019, and by 2023, more than 5 million cases and approximately 5000 deaths attributable to this disease had been recorded [14,15].

In the Americas, Mexico reports the highest number of dengue cases [10,11], with transmission primarily associated with *Ae. aegypti*. Historically, dengue transmission has been reported in 28 of the 32 states of the Mexican Republic; however, currently, only two states (Mexico City and Tlaxcala) are considered free of endemic transmission [16,17]. It should also be noted that the clinical classification of dengue in Mexico has evolved over time. Between 1990 and 2007, the Sistema Nacional de Vigilancia Epidemiológica (SINAVE) classified the disease as classic dengue and hemorrhagic dengue [18]. From 2008 to 2016, the terms dengue fever and dengue hemorrhagic fever were used [19]. Since 2017, the classification proposed by the WHO and the Pan American Health Organization (PAHO) has been adopted, distinguishing between non-severe dengue (NSD), dengue with warning signs (DWS), and severe dengue (SD) [20].

Climate change is considered a determining factor in the geographical expansion and increased incidence of dengue. Sustained increases in temperature, changes in precipitation patterns, and higher environmental humidity create favorable conditions for the proliferation and survival of *Ae. aegypti*, the main vector of the virus. These environmental changes may expand endemic areas and prolong transmission periods, even in regions where the risk was previously minimal. Temperature is a critical determinant of dengue transmission because it directly influences mosquito development, survival, biting frequency, and the extrinsic incubation period of the virus [21,22,23]. However, dengue dynamics arise from a multifactorial system that also includes precipitation patterns, humidity, urbanization, human mobility, vector control strategies, population immunity, and viral serotype circulation [21,22,23,24]. Therefore, temperature should be interpreted as one important climatic component within a broader transmission context rather than a proxy for all environmental and biological drivers. The aim of this study was to describe national dengue case trends in Mexico from 1990 to 2023 and to assess their association with temperature over the same period using a descriptive, retrospective analysis of epidemiological surveillance and temperature data.

## 2. Materials and Methods

### 2.1. Epidemiological Data Collection

Information was collected in accordance with the principles of confidentiality and discretion set forth in the Federal Law on Transparency and Access to Public Information. Epidemiological data were taken from the morbidity yearbook (1990–2023) of the General Directorate of Epidemiology, available at https://epidemiologia.salud.gob.mx/anuario/html/index.html (accessed on 25 August 2025). Only laboratory-confirmed dengue cases are included in these official reports. The information reported on this platform has been previously generated and analyzed by the Ministry of Health through its website www.sinave.gob.mx (accessed on 27 August 2025). No ethical approval was obtained for the use of epidemiological information on confirmed cases and incidence dengue in Mexico.

### 2.2. Operational Definitions of Case Dengue in Mexico from 1990 to 2023

For this study, dengue case classification based on historical operational definitions was not reanalyzed. Instead, we used the total annual number of dengue cases and incidence as reported in the Official Morbidity Yearbook of the General Directorate of Epidemiology of the Mexican Ministry of Health for the period 1990–2023. These totals correspond to cases considered laboratory-confirmed in the national surveillance system for each year, regardless of changes in operational case definitions over time [18,19,20].

The operational definitions presented below summarize the official criteria used by the Mexican epidemiological surveillance system during each period.

#### 2.2.1. Operational Definitions of Dengue Case from 1990 to 2007

During the period 1990–2007, the Sistema Nacional de Vigilancia Epidemiológica (SINAVE) classified dengue cases into classic dengue and dengue hemorrhagic fever.

Suspected dengue case: Any person presenting with nonspecific fever consistent with an endemic viral infection. Probable classic dengue case: Any suspected case presenting with fever accompanied by headache, myalgia, and arthralgia.

Probable dengue hemorrhagic fever case: Any person initially presenting with classic dengue who subsequently developed clinical signs compatible with severe disease, including persistent fever, evidence of plasma leakage, capillary fragility or hemorrhagic manifestations, thrombocytopenia, and hemoconcentration.

Confirmed case: Any suspected or probable case with recent dengue virus infection confirmed by laboratory techniques or epidemiologically linked to another laboratory-confirmed case.

#### 2.2.2. Operational Definitions of Dengue Case from 2008–2016

Between 2008 and 2016, the surveillance system maintained the distinction between dengue fever and dengue hemorrhagic fever but introduced more detailed clinical criteria.

A probable dengue fever case was defined as a suspected case presenting with fever and at least two additional symptoms such as headache, myalgia, arthralgia, rash, or retro-ocular pain. In children under five years of age, fever alone could be considered sufficient for suspicion.

Dengue hemorrhagic fever was defined as a probable dengue fever case presenting additional evidence of plasma leakage, capillary fragility or bleeding manifestations, thrombocytopenia (<100,000 platelets/mm^3^), or hemoconcentration. Confirmed cases required laboratory confirmation or epidemiological linkage with a laboratory-confirmed case.

#### 2.2.3. Operational Definitions of Dengue for 2017–2023

From 2017 onward, Mexico adopted the World Health Organization (WHO) and Pan American Health Organization (PAHO) dengue classification system.

Under this framework, dengue cases are categorized as:

Non-severe dengue (NSD): Fever with at least two symptoms such as nausea or vomiting, rash, myalgia or arthralgia, headache or retro-ocular pain, petechiae, or leukopenia in individuals living in or traveling from endemic areas.

Dengue with warning signs (DWS): NSD cases presenting additional clinical warning signs including persistent abdominal pain, persistent vomiting, mucosal bleeding, fluid accumulation, lethargy or irritability, hepatomegaly, increasing hematocrit, or decreasing platelet counts.

Severe dengue (SD): Cases with severe plasma leakage leading to shock or respiratory distress, severe bleeding, or severe organ involvement (e.g., liver, kidney, central nervous system, or cardiac complications).

Confirmed cases in all categories require laboratory confirmation by diagnostic methods recognized by the Instituto de Diagnóstico y Referencia Epidemiológicos (InDRE). Probable cases in which infection is not confirmed are classified as discarded cases.

### 2.3. National Temperature Analysis

Variations in the national average temperature were obtained from monthly reports issued by the National Water Commission (CONAGUA), available at https://smn.conagua.gob.mx/es/climatologia/temperaturas-y-lluvias/resumenesmensuales-de-temperaturas-y-lluvias (accessed 25 August 2025). Annual mean temperatures for Mexico from 1990 to 2023 were calculated from these records. Statistical analyses and graphical visualizations were performed using GraphPad Prism version 10.3.1. (GraphPad Software, San Diego, CA, USA) Temporal trends in temperature were evaluated by constructing a time-series graph for the study period (1990–2023).

To identify statistically distinct temporal phases in national temperature trends, a one-way ANOVA followed by a Tukey post hoc test was performed. Statistical significance was established at *p* ≤ 0.01. This analysis allowed the identification of statistically significant differences in temperature trends across the study period and supported the classification of two temporal phases.

### 2.4. Total Cases and Incidence of Dengue

All total cases and incidence of dengue, without restriction of age or sex, reported in the 32 states of the Mexican Republic from 1990 to 2023 issued by SINAVE were included. Epidemiological data for 2024 are not yet available on the website consulted. Mexican regulations establish that it is mandatory to report confirmed cases of dengue in the 32 states that make up the Mexican territory. The information reported on this platform has been previously generated and analyzed by the Ministry of Health through its website www.sinave.gob.mx (accessed on 25 August 2025).

### 2.5. Analysis of the Incidence and Total Cases of Dengue in Mexican Territory

The incidence of dengue (per 100,000 inhabitants) and the total number of cases reported by the GDE in the 32 states of the Mexican Republic during the period between 1990 and 2023 were analyzed annually. The population estimates used as the denominator for incidence calculations were obtained from official population projections prepared by the El Consejo Nacional de Población (CONAPO), which are based on national census data and intercensal demographic estimates and provide annual population estimates at the national and subnational levels. The incidence of dengue was calculated by dividing the number of reported cases by the estimated population for the same geographic area and the same year and is expressed per 100,000 inhabitants. With this information, epidemic curves of total cases and incidence of dengue infection were constructed to describe the behavior of these two epidemiological indicators in the population. Student’s *t*-test was performed to determine whether there were significant differences in total cases/incidence. GraphPad Prism 10.3.1 was used for analysis and visualization.

### 2.6. Geographic Distribution of Dengue Incidence in Mexican Territory

An incidence analysis by geographic distribution was performed, considering the 32 states of the Mexican Republic and grouped into quartiles, with quartiles 4 (Q4) and 1 (Q1) having the highest and lowest incidence, respectively. The analysis and visualization were performed using Microsoft Excel (Microsoft Corporation, Redmond, WA, USA).

### 2.7. Analysis of Incidence Increment of Dengue in Mexican Territory

With the aim of identifying states that showed a significant increase in the incidence of dengue, Student’s *t*-test (*p* < 0.05) was performed on the incidence in all states of the Mexican territory. The Mexican states where a significant difference in the increase in incidence was observed were selected, and GraphPad Prism 10.3.1 was used for analysis and visualization.

### 2.8. Association Between Temperature and Dengue Cases

To evaluate the relationship between temperature and dengue trends, two complementary analyses were performed to evaluate both national temporal trends and potential geographic variability in the temperature–dengue relationship.

First, a national-level analysis was conducted to assess the association between annual dengue trends and national average temperature. Pearson correlation analysis was performed between the total number of dengue cases and dengue incidence reported annually from 1990 to 2023 and the corresponding national mean temperature for each year. Statistical analysis and graphical visualization were performed using GraphPad Prism version 10.3.1.

Second, to explore potential geographic variability in the association between temperature and dengue incidence, an additional state-level analysis was performed in the same analysis period (1990–2023). Annual mean temperature data for each Mexican state were obtained from the records of the Comisión Nacional del Agua (CONAGUA) for the period 1990–2023. These temperature data were linked with the corresponding average dengue incidence values by state for the period.

The association between mean temperature and dengue incidence at the state level was evaluated using Pearson correlation analysis, and graphical representations were generated using GraphPad Prism version 10.3.1. Linear regression lines with 95% confidence bands were included in the graphical representations.

### 2.9. K-Medoids Analysis

In order to identify regional transmission patterns, a partitioning cluster analysis was applied using the k-medoids algorithm, a technique that is robust to outliers and suitable for epidemiological data with a non-normal distribution. Unlike k-means, which uses means as centroids, k-medoids uses actual observations (medoids) as cluster centers, allowing for greater stability and representativeness of the groups. The optimal number of clusters was defined in three categories (low, medium, and high incidence variability), based on epidemiological criteria and the consistency of the classification with the observed distribution of the data.

#### Dimensionality Reduction and Visualization

Principal Coordinate Analysis (PCoA) was used to graphically represent the results based on the dissimilarity matrix. The first two axes (Dim1 and Dim2) explained the highest percentage of variability between entities and were used to project the spatial distribution of clusters in each period. For the statistical analysis of the data, RStudio (version 2025.05.1+514) was used, along with the cluster and factoextra packages. The cluster graphs were generated with the multivariate visualization functions available in these packages.

## 3. Results

### 3.1. Analysis of Temperature Behavior in Mexican Territory

**Figure 1:**
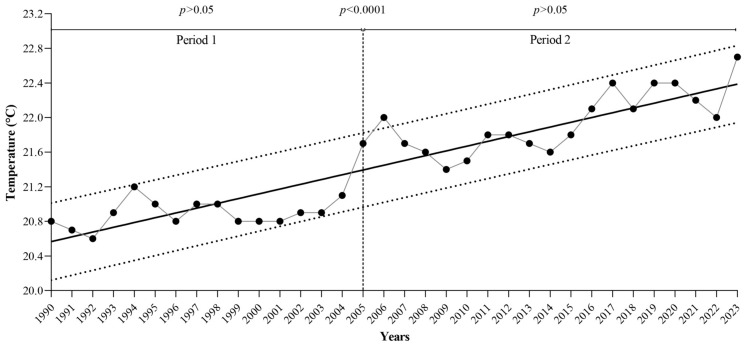
Temporal behavior of temperature in Mexican territory from 1990 to 2023. Two statistically significant periods (*p* < 0.0001) and an upward trend over the years are observed.

To explore the temporal behavior of temperature, a one-way analysis of variance (ANOVA) followed by a Tukey post hoc test was performed to evaluate differences in the mean annual temperature across the study years (Figure 1). The analysis showed no statistically significant differences in temperature during the first 16 years of the study period (1990–2005) at the national level. However, significant differences were observed when these years were compared with the subsequent 18-year period (2006–2023). No significant differences were detected within the latter period.

Based on these findings, the study analysis was divided into two time periods (1990–2005 and 2006–2023), reflecting the significant change in temperature observed in Mexico during the study period.

### 3.2. Epidemiological Behavior of Total Cases and Incidence of Dengue from 1990 to 2023

**Figure 2:**
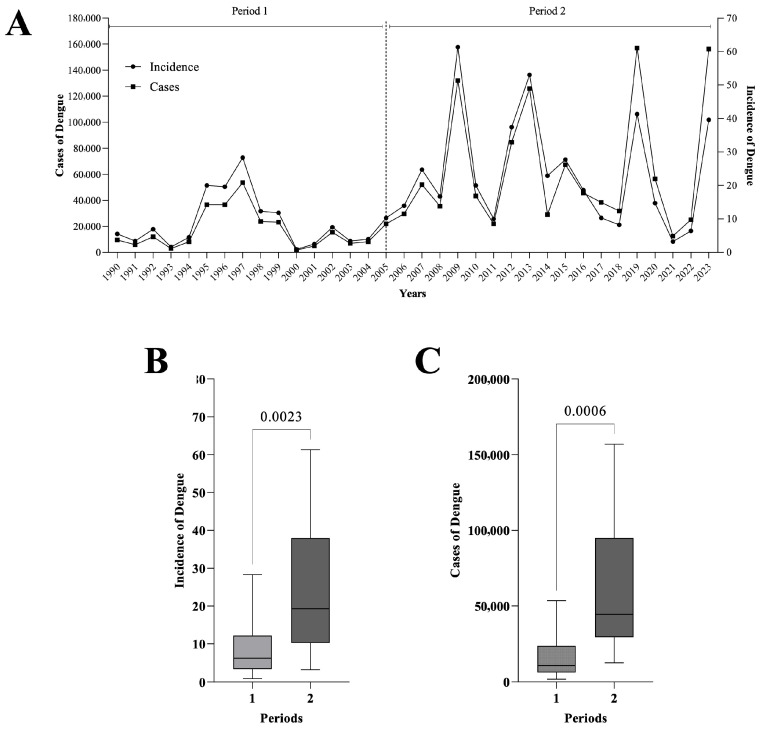
Epidemiological behavior of dengue in Mexico from 1990 to 2023. (**A**): Epidemic curves of total cases/incidence of dengue, (**B**): Student’s *t*-test of the incidence of dengue in the two periods, (**C**): Student’s *t*-test of total cases of dengue in the two periods.

The separation into two periods (1990–2005, 2006–2023) due to the temperature behavior described above showed an upward trend in total cases/incidence, with a higher number of cases (total/incidence) observed in period 2 (Figure 2A). The above was validated by statistical analysis (Student’s *t*-test) of total cases/incidence (Figure 2B,C).

### 3.3. National Geographic Distribution of Dengue Incidence

**Figure 3:**
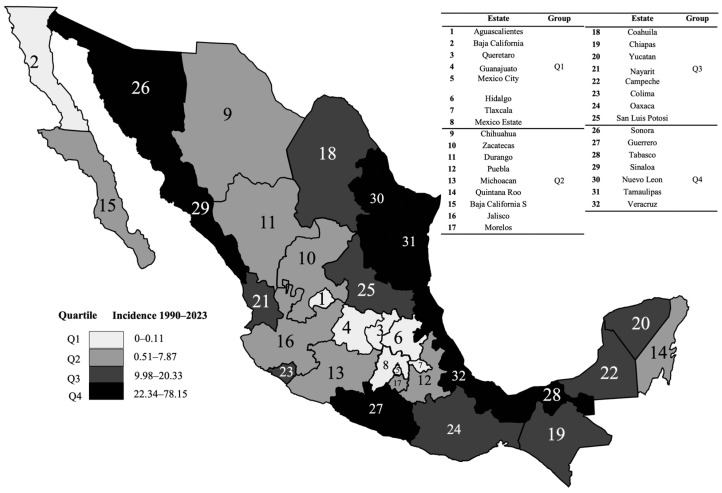
Geographic distribution of incidence rates of confirmed cases dengue (per 100,000 inhabitants) in Mexico during 1990–2023.

To understand the geographical distribution of dengue incidence at the national level, a heat map of the epidemiological distribution was generated. This analysis allowed the integration of four quartiles (Q1–Q4) (Figure 3).

### 3.4. Increase in the Incidence of Dengue by State

**Figure 4:**
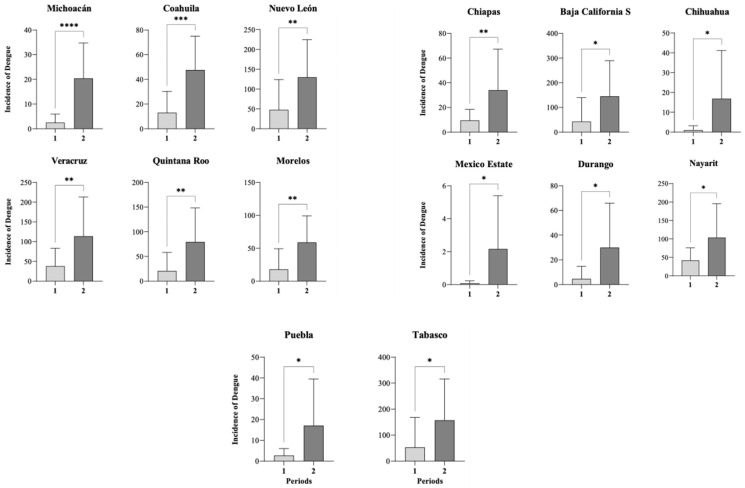
Increase in the incidence of dengue in Mexico states compared between two periods: period 1 (1990–2005) and period 2 (2006–2023). Significance level * *p *< 0.05, ** *p *< 0.001, *** *p *< 0.0001, **** *p* < 0.00001.

The results showed that the increase in incidence was significant between periods in 14 of the 32 states: Baja California Sur, Chihuahua, Coahuila, Durango, Nuevo León, Michoacán, Nayarit, Estado de México, Puebla, Morelos, Veracruz, Tabasco, Chiapas, and Quintana Roo (Figure 4).

### 3.5. Correlation of Incidence of Dengue and Temperature

**Figure 5:**
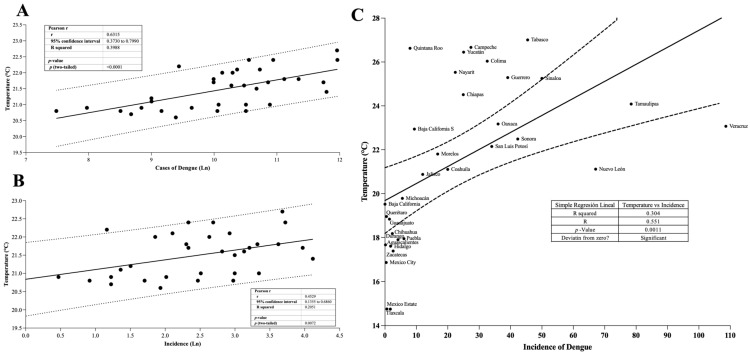
Correlation between temperature and dengue trends in Mexico from 1990 to 2023. (**A**): Relationship between mean annual temperature and total dengue cases. (**B**): Relationship between mean annual temperature and dengue incidence. (**C**): Association between mean state temperature and mean dengue incidence across the 32 Mexican states. Each point represents one state. Solid lines indicate linear regression models, and dashed lines represent 95% confidence bands.

Figure 5A shows the correlation between temperature and the number of national dengue cases with a 95% CI. Pearson’s correlation analysis determined an r = 0.6315, showing a strong correlation. On the other hand, Figure 5B also shows the correlation between temperature and incidence, with the test determining an r of 0.4529.

An additional analysis was performed to evaluate the relationship between the mean temperature and dengue incidence at the state level (Figure 5C). Pearson correlation analyses showed a positive association between temperature and dengue incidence in the analyzed period (1990–2023). Overall, states with higher mean temperatures tended to present higher dengue incidence values.

The regression analysis confirmed the general pattern observed in the national-level analysis.

### 3.6. Incidence Behavior in Mexican Territory

Cluster analysis using the k-medoids algorithm identified three distinct groups of states according to the incidence of dengue in Mexico, both in the first period (1990–2005) and in the second (2006–2023). In both cases, the clusters were interpreted based on the variation in incidence among states grouped into low, medium, and high incidence variability. In the first period (1990–2005), the two-dimensional representation showed that Dimension 1 revealed 28.4% of the variability and Dimension 2 revealed 15.1%. Most entities were grouped in the low and medium incidence variability clusters, while a small number of states were in the high incidence variability cluster. It is noteworthy that several endemic entities, such as Quintana Roo, Yucatan, Veracruz, Chiapas, and Tabasco, were positioned in the medium incidence group, because their endemicity causes dengue transmission to be sustained, but without the epidemic peaks observed in other regions grouped in the high variability cluster. In the second period (2006–2023), a more defined separation between clusters was observed compared to the previous period. Dimension 1 explained 37.4% of the variability and Dimension 2 explained 13.6%, demonstrating the model’s greater discriminatory capacity. During this period, multiple entities moved toward the medium incidence cluster, which includes most of the states considered endemic, reflecting an increase in the frequency of outbreaks compared to the first period. It should be noted that the vectors corresponding to states located in the low incidence cluster (Yucatán, Oaxaca, Morelos, Quintana Roo, and Chiapas) are moving toward the medium incidence cluster, suggesting an upward trend in transmission variability, despite their initial classification. Likewise, the dispersion of points within the multivariate space reveals greater regional heterogeneity, with some states maintaining low transmission patterns and others experiencing recurrent epidemics of high intensity.

## 4. Discussion

Dengue has become an increasing public health concern in Mexico, with rising temperatures associated with climate change identified as a key driver of its transmission dynamics [5,22,23,25,26,27,28].

The findings of this study demonstrate a significant increase in national temperature over the study period, with a clear transition between 1990–2005 and 2006–2023 (Figure 1). Similar warming trends have been documented in previous studies, highlighting the progressive increase in temperature and its broad environmental consequences [29,30,31]. From a public health perspective, rising temperatures are particularly relevant for vector-borne diseases, as they influence mosquito survival, reproduction, and geographic distribution, thereby facilitating pathogen transmission in tropical and subtropical regions [29].

**Figure 6:**
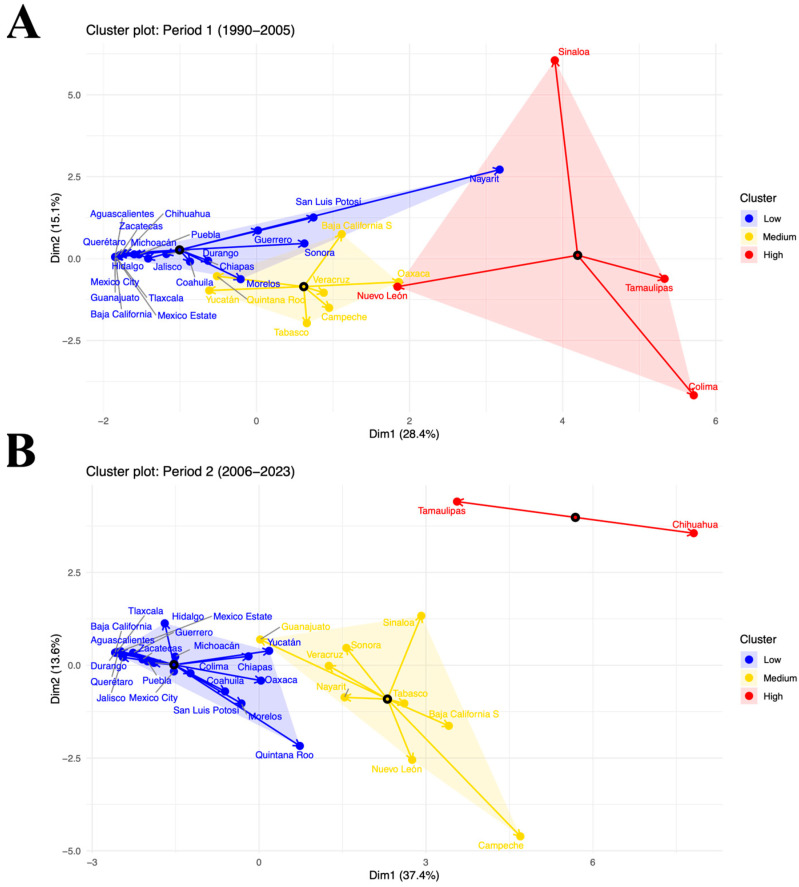
The k-medoids analysis produced a cluster plot of dengue incidence in Mexico. (**A**): Period 1 (1990–2005): two major components are represented by the x (dimension 1) and y (dimension 2) axes, which account for 15.1% and 28.4% of the variation in the data, respectively. (**B**): Period 2 (2006–2023): two major components are represented by the x (dimension 1) and y (dimension 2) axes, which account for 13.6% and 37.4% of the variation in the data, respectively.

Our results revealed a significant increase in total dengue cases and incidence in Mexico during the study period (Figure 2, Figure 3 and Figure 4). Despite the implementation of national prevention programs for vector-borne diseases [32], the findings suggest that dengue transmission has continued to expand. Furthermore, dengue incidence showed a positive association with the increase in temperature observed during the same period (Figure 5). Similar relationships between increasing temperatures and dengue transmission have been reported in other regions of the world [33]. A study conducted in Mexico reported 1230 dengue-related deaths between 2007 and 2020, corresponding to the second temporal period identified in this study, during which dengue incidence increased significantly. The highest mortality figures were recorded in 2009, 2012, 2013, and 2019, coinciding with years in which high numbers of dengue cases and incidence were observed (Figure 2) [25]. The statistical analysis revealed that several states, including Baja California Sur, Chihuahua, Coahuila, Durango, Nuevo León, Michoacán, Nayarit, Estado de México, Puebla, Morelos, Veracruz, Tabasco, Chiapas, and Quintana Roo, showed significant increases in dengue incidence between the two periods analyzed (Figure 4). States such as Guerrero, Tabasco, and Veracruz (grouped in Q4), as well as Chiapas, Yucatán, and Campeche (grouped in Q3), represent dengue transmission hot spots (Figure 3) [34]. In contrast, some regions that were not previously considered transmission hot spots showed increasing incidence, suggesting a possible expansion of dengue risk areas. Multiple factors may contribute to this expansion. Climatic conditions such as increased temperature and rainfall may favor mosquito proliferation, while anthropogenic factors including water storage practices, inadequate waste management, and population mobility may also contribute to the spread of dengue virus and the introduction of new viral serotypes in affected regions [35,36,37,38]. The K-medoids clustering analysis (Figure 6) revealed an epidemiological transition in dengue distribution in Mexico. During the period 1990–2005, dengue incidence was concentrated in a limited number of states, whereas between 2006 and 2023, the disease showed a broader and more intense distribution across the country. This clustering reflects patterns of incidence variability and does not imply that temperature alone explains these regional groupings. Instead, the observed patterns are likely influenced by a combination of climatic conditions, urbanization processes, and the expansion of the mosquito vector [39].

In Mexico, the primary vector responsible for dengue transmission is *Ae. aegypti*. However, the presence of this mosquito has recently been reported in regions where it was previously absent, such as Mexico City, where eggs have been detected since 2015 [8,40]. Similar observations have been reported in other regions of the world, where climatic changes have facilitated the expansion of this vector into new geographic areas [41,42,43]. The detection of dengue cases in previously non-endemic regions highlights the potential risk of future outbreaks in areas where the vector becomes established [44,45,46]. In 2024, the CDC (Centers for Disease Control and Prevention) implemented guidelines for responding to dengue cases in non-endemic areas in the United States and recognized Mexico as a country with a high risk of dengue transmission [47,48]. The growing burden of dengue also has implications for health systems, including blood safety. Cases of dengue transmission through contaminated blood products have been reported, resulting in infections and fatalities [49,50]. In Mexico, the NOM-253-SSA1-2012 regulation governing the use of human blood and its components for therapeutic purposes does not currently require mandatory dengue screening, even in endemic regions [51]. Given the increasing incidence of dengue in the country, strategies aimed at strengthening blood safety should be considered.

In response to the increasing incidence of dengue and other arboviral diseases, Mexico has implemented a National Plan for the Control of Dengue and Other Arboviruses [52]. This strategy includes epidemiological surveillance, vector control, community participation, and scientific innovation. Currently, mosquito density is monitored through more than 220,000 ovitraps distributed across 30 states, 364 municipalities, and 712 localities, allowing the identification of high-risk areas and the implementation of targeted prevention strategies [52]. Additional control measures include the use of larvicides, long-lasting insecticides, and fogging in strategic areas [53]. Community participation also plays a crucial role in eliminating mosquito breeding sites in homes and public spaces, contributing to the prevention of dengue transmission [54].

Temperature is a critical environmental determinant of mosquito biology and vectorial capacity. In *Ae. aegypti* and *Ae. albopictus*, temperature influences multiple physiological and ecological processes, including larval development rate, adult survival, reproductive capacity, and biting frequency. Warmer temperatures can also shorten the extrinsic incubation period of the dengue virus within the mosquito, allowing infected vectors to become infectious more rapidly and potentially increasing transmission efficiency. These temperature-dependent biological processes provide a mechanistic basis for understanding how climatic conditions may influence dengue transmission dynamics at the population level [22].

Overall, the results of this study demonstrate a sustained increase in dengue cases and incidence in Mexico over time, as well as a positive association between dengue transmission and increasing national temperature. These findings highlight the importance of considering climatic factors, particularly temperature trends associated with climate change, when evaluating the future dynamics and geographic expansion of dengue in Mexico.

## 5. Limitations

A limitation of this study is its design, which precludes causal inference. Although a positive association was observed between temperature and dengue incidence/case numbers, this relationship should not be interpreted as evidence that temperature alone drives dengue transmission dynamics. Dengue epidemiology is inherently multifactorial, and the observed trends likely reflect the combined effects of climatic, biological, social, and historical processes. The Americas, including Mexico, are predominantly tropical and subtropical regions where dengue virus transmission and the establishment of *Aedes aegypti* have been historically favored. The progressive geographic expansion and consolidation of transmission cycles over time, following repeated epidemic introductions, may have contributed substantially to the increasing magnitude of dengue epidemics observed during the study period, independently of recent temperature changes.

In addition, this study did not include entomological data, such as vector density or classical infestation indices. Other climatic variables known to influence mosquito reproduction and survival, including rainfall and humidity, were not analyzed. Likewise, information on relevant socio-epidemiological factors was not available, including urbanization patterns, human mobility, changes in vector control strategies, viral serotype circulation, and temporal variations in surveillance sensitivity, all of which could confound or mediate the observed association between temperature and reported dengue cases. Finally, the use of national annual mean temperature may obscure important regional and seasonal variability in both climate and dengue transmission, particularly in a geographically heterogeneous country such as Mexico. The absence of spatially disaggregated or seasonal analyses limits the ability to identify localized dynamics and differential regional effects. Taken together, these limitations indicate that while temperature is a relevant variable for vector survival and dengue transmission, it should be interpreted as a contributing factor within a complex system rather than as the primary or exclusive driver of national-level dengue trends.

## 6. Conclusions

This study describes a long-term increase in dengue cases and incidence in Mexico during the analyzed period and identifies a temporal association between dengue trends and increasing national average temperature. Although temperature is a relevant factor influencing vector survival and transmission potential, dengue dynamics arise from a complex interaction of climatic, biological, and socio-epidemiological processes. Therefore, given the nature of this analysis, these findings should be interpreted as exploratory and hypothesis-generating rather than as evidence of a causal relationship, and the observed trends should not be attributed solely to temperature. In this context, multifactorial epidemiological surveillance is essential to strengthen early-warning systems and to support timely and effective dengue prevention and control strategies in both endemic and non-endemic areas, particularly in scenarios of environmental and demographic change.

## Data Availability

The data that support the findings of this study are openly available in Mendeley Data at https://data.mendeley.com/datasets/ry25nh9h2m/1. (accessed on 25 August 2025).
